# The Immune Tolerance Role of the HMGB1-RAGE Axis

**DOI:** 10.3390/cells10030564

**Published:** 2021-03-05

**Authors:** Haruki Watanabe, Myoungsun Son

**Affiliations:** 1 Center for Autoimmune Musculoskeletal and Hematopoietic Diseases, The Feinstein Institutes for Medical Research, 350 Community Drive, Manhasset, NY 11030, USA; hwatanabe@northwell.edu; 2 Department of Molecular Medicine, Zucker School of Medicine at Hofstra/Northwell, Hempstead, NY 11549, USA

**Keywords:** high mobility group box 1, receptor for advanced glycation endproducts, lupus, hypoxia, inflammation, tolerance

## Abstract

The disruption of the immune tolerance induces autoimmunity such as systemic lupus erythematosus and vasculitis. A chromatin-binding non-histone protein, high mobility group box 1 (HMGB1), is released from the nucleus to the extracellular milieu in particular environments such as autoimmunity, sepsis and hypoxia. Extracellular HMGB1 engages pattern recognition receptors, including Toll-like receptors (TLRs) and the receptor for advanced glycation endproducts (RAGE). While the HMGB1-RAGE axis drives inflammation in various diseases, recent studies also focus on the anti-inflammatory effects of HMGB1 and RAGE. This review discusses current perspectives on HMGB1 and RAGE’s roles in controlling inflammation and immune tolerance. We also suggest how RAGE heterodimers responding microenvironments functions in immune responses.

## 1. Immune Tolerance and Autoimmunity

Failure of immune tolerance results in lymphocyte reactions against self-antigens called autoimmunity and the diseases caused by autoimmunity are referred to as autoimmune diseases. Autoimmune reactions are triggered by environmental factors, such as infections, in genetically susceptible individuals. Autoimmune diseases are classified as a systemic or organ-specific disease, depending on the distribution of the autoantigens that are recognized [1]. For example, autoantibodies against ubiquitous antigens, including nuclear components typically cause systemic diseases, such as systemic lupus erythematosus (SLE) and vasculitis. Autoantibodies or T cell responses against self-antigens with restricted tissue distribution lead to organ-specific diseases, such as autoimmune thyroiditis, type 1 diabetes, and myasthenia gravis. The adaptive immune response including autoreactive T lymphocytes, circulating autoantibodies and immune complexes is generally thought to be responsible for tissue injury in autoimmune diseases.

Most autoimmune diseases are polygenic, and numerous susceptibility genes contribute to the predisposition to disease development. SLE is a systemic disease in which autoantibodies such as anti-DNA antibodies form immune complexes and is characterized by its heterogeneous clinical manifestations, including cutaneous, kidney, central nervous system [2,3,4]. Though the pathogenesis of SLE has not been fully elucidated, there is abnormal clearance of apoptotic debris which induce anti-DNA antibody production and lead to inflammation causing clinical symptoms [5]. It frequently occurs in young women, and various immunosuppressive drugs are used for treatment [6]. The impact of genetic susceptibility on the development of SLE has been well demonstrated in a number of large-scale genome-wide association studies (GWAS) [7]. Notably, a functional SNP located in the 3′ untranslated region of TLR7 was associated with SLE. The Interferon regulatory factor 5 (IRF5) genetic locus carries multiple functional polymorphisms that potentially associate with SLE [8]. Genetic deficiency of C1q predisposes strongly to SLE and C1q polymorphisms are associated with more severe SLE, low serum C1q, and low levels of total hemolytic complement [9].

Antineutrophil cytoplasmic antibody (ANCA)-associated vasculitis (AAV) is another systemic autoimmune disease. AAV is characterized by ANCA production and small- and medium-sized blood vessel inflammation [10,11]. AAV commonly causes life-threatening kidney failure or pulmonary hemorrhage and affects short- and long-term mortality. GWAS has revealed that HLA, α1-antitrypsin and proteinase 3 are associated with ANCA specificity [12].

Several tolerance mechanisms have been studied in monocytes/macrophages contributing to scavenging, inflammation, and anti-pathogen defenses [13,14,15]. Ineffectual clearance of immune complexes and accumulation of apoptotic cells expose the immune system to various autoantigens [7]. The repeated or chronic activation of Toll-like receptors (TLRs) by a bacterial product such as lipopolysaccharide (LPS) induce immune tolerance to the secondary infection [16,17,18]. These mechanisms are important to prevent prolonged or repeated activation of TLRs leading to uncontrolled inflammation and subsequent damages.

Macrophage polarization is another way which maintains immune homeostasis. Monocytes can be polarized through different activation programs to the classical pro-inflammatory M1 macrophages and the anti-inflammatory M2 macrophages [19,20]. Pro-inflammatory macrophages contribute to immune protection in infection, but also to disease pathogenesis in autoimmune diseases, atherosclerosis and Alzheimer’s disease and anti-inflammatory macrophages are important for cessation of inflammation and tissue repair.

High mobility group box 1 (HMGB1) is a damage-associated molecular pattern (DAMPs) and high in autoimmune diseases, tissue injury and infection [2,21,22]. HMGB1 exhibits many other functions in immune response, either pro-inflammatory or anti-inflammatory. In this review, we describe the role of HMGB1 as a positive and negative regulator for inflammatory autoimmune diseases. We describe the tolerance mechanism of HMGB1 that provides potential therapeutics for autoimmune diseases.

## 2. HMGB1

**Figure 1:**
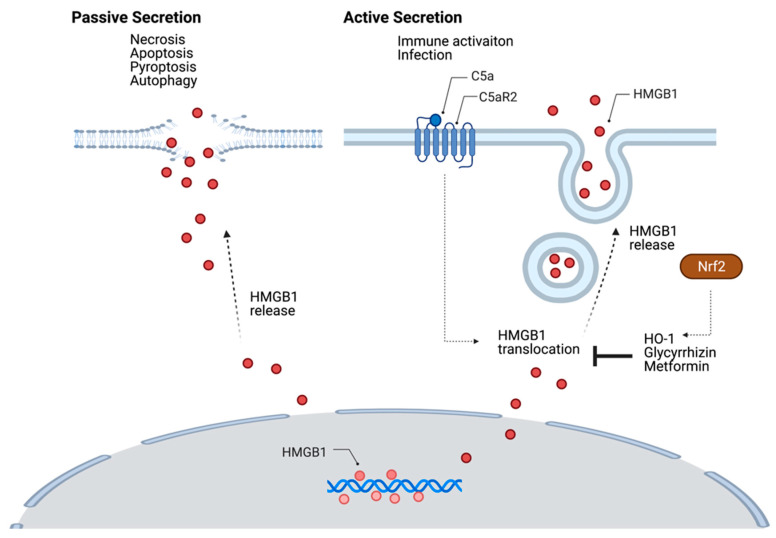
The regulation of HMGB1 secretion. Passive release of HMGB1 involves the plasma membrane disruption through cell death mechanisms. Inflammation and immune activation induce HMGB1 secretion. Post-translational modifications-mediated active secretion of HMGB1 occurs via secretory lysosomes. C5a and C5aR2 pathway induces HMGB1 release. Small molecules such as glycyrrhizin and metformin inhibit nuclear HMGB1 translocation to the cytosol. The nuclear factor-erythroid 2-related factor 2 (Nrf2)/hemeoxygenase-1 (HO-1) pathway also suppresses the translocation and secretion of HMGB1.

HMGB1 is an evolutionary conserved nuclear protein that binds to DNA to maintain chromatin structure, involves DNA repair, and indirectly regulates the activities of various transcription factors such as NF-κB and glucocorticoid receptors [23,24]. HMGB1 is highly conserved in mammals [25]. HMGB1 has 215 amino acid residues and forms two DNA binding domains (A box [9–79 amino acid], B box [95–163 amino acid]) and a C-terminal acidic tail (186–215 amino acid) [26]. HMGB1 is released passively from necrotic cells or actively from activated dendritic cells and macrophages, then relates to various pathological conditions [27]. Caspase 3/7-mediated programmed cell death, autophagy, pyroptosis cause the passive release of HMGB1. Active secretion of HMGB1 occurs through the extensive post-translational modifications (PTM) such as acetylation, phosphorylation, methylation and oxidation that determine the localization of HMGB1. PTM-mediated active secretion of HMGB1 is mediated through secretory lysosomes [28]. The oxidation status of HMGB1 and HMGB1 secretion kinetics also influences its immune function [28]. Although the exact active secretion mechanism of HMGB1 remains elusive, a recent study has revealed that C5a engagement with its receptor C5aR2 in macrophages upon infection induces upregulation of HMGB1 expression and release through intracellular signaling [29] (Figure 1).

Small molecules can inhibit HMGB1 secretion [28]. Glycyrrhizin inhibits the cytoplasmic transduction of HMGB1 and suppresses the expression of inflammatory cytokines [28]. Anti-inflammatory drug, Metformin binds HMGB1 and inhibits nuclear HMGB1 translocation to the cytosol [30,31]. The nuclear factor-erythroid 2-related factor 2 (Nrf2)/hemeoxygenase-1 (HO-1) pathway also plays an important role in the HMGB1 secretion. HO-1 suppresses the translocation and secretion of HMGB1 [32,33]. Nrf2 is a redox-sensitive transcription factor for HO-1 [34,35] (Figure 1). Anti-inflammatory vitamin D (1,25-dihydroxy vitamin D) inhibits LPS-induced HMGB1 secretion in macrophages through the Nrf2/HO-1 pathway [34].

Extracellular HMGB1 can bind with the receptor for advanced glycation endproducts (RAGE), TLRs, and cytosolic DNA/RNA sensors mediating inflammation [36,37]. Disulfide HMGB1 binds to MD2 in the TLR4 receptor complex and induces cytokine production [38]. All HMGB1 redox forms bind to RAGE and HMGB1-RAGE interaction results in uptake in endosomes that present TLR7 and 9 [39]. Without any doubt, HMGB1 is a crucial mediator for the innate immune system and an attractive target for therapy in many disease states, including sepsis, ischemia, arthritis, autoimmune diseases, neurodegenerative diseases, metabolic disorders and cancer [40]. Gain- and loss-of-function analysis showed that HMGB1 exacerbated the severity of renal disease and autoimmunity in the murine model of SLE [41]. Extracellular blockades such as neutralizing mouse/rat/humanized anti-HMGB1 antibodies, receptor blocking HMGB1 A box and FSSE tetramer have the distinct potential to improve clinical outcome in multiple inflammatory diseases [38,42,43,44].

Through extensive studies by others, extracellular HMGB1 is highly inclined to bind many molecules, and most other receptors are presented in a complex form with HMGB1. Besides, RAGE has multi ligands. Current studies suggest that the alternative mechanistic explanation for HMGB1 and RAGE axis is that this interaction provides both pro-inflammatory and anti-inflammatory functions—the critical functional difference may be caused by HMGB1-RAGE attached to partner molecules.

## 3. RAGE Functions Following HMGB1 Engagement

**Figure 2:**
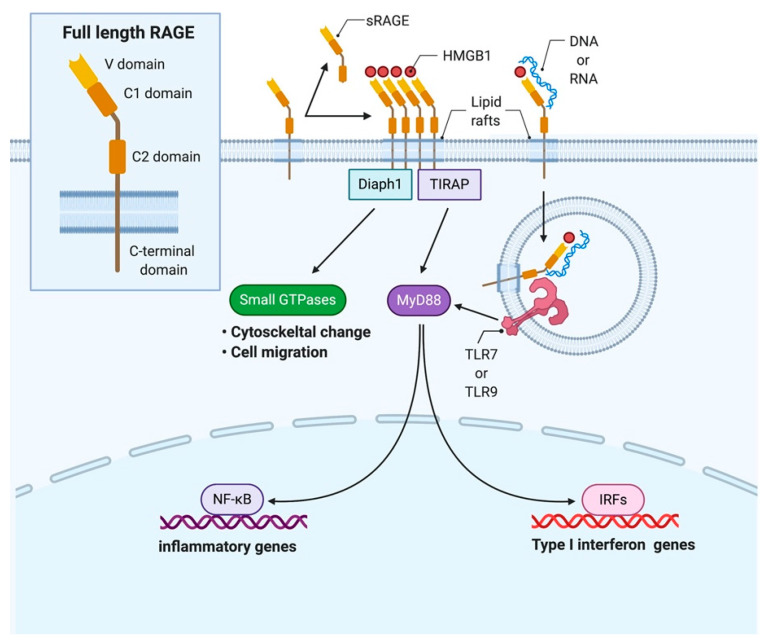
RAGE structure and signaling pathway. The receptor for advanced glycation endproducts (RAGE) has three extracellular immunoglobulin-like domains (V, C1, and C2), a single transmembrane helix, and a C-terminal short domain, and exists in lipid rafts. Soluble RAGE (sRAGE) is created by alternative splicing or cleaved by protease and functions as a decoy receptor. When binding with high mobility group box 1 (HMGB1) and nucleic acid, RAGE internalizes into the cytosol and interacts with Toll-like receptors (TLRs). RAGE transduces down-stream signaling upon binding with ligands and adaptor proteins, including diaphanous homolog 1 (Diaph1), toll-interleukin 1 receptor domain-containing adaptor protein (TIRAP), and myeloid differentiation primary response 88 (MyD88), resulting in type 1 interferon and pro-inflammatory cytokines production.

**Figure 3:**
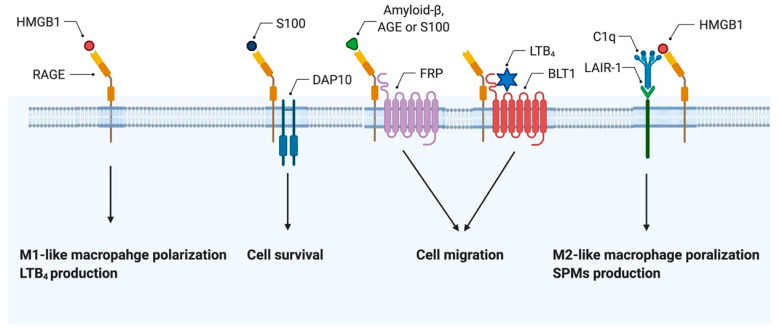
RAGE function as heterodimers. The functional interaction between RAGE and DNAX-activating protein 10 (DAP10) coordinately regulates S100-mediated cell survival. Formyl-peptide-receptors (FPRs) reacts with the broad ligand spectrum through the interaction with RAGE. Leukotriene B_4_ (LTB_4_) receptor 1 (BLT1) interacts with RAGE and induces proinflammatory cytokines and chemokines. HMGB1 promotes leukotriene production, induces interferon regulatory factor 5 in a RAGE-dependent manner. C1q can form a multimolecular signaling complex with HMGB1, RAGE, and Leukocyte-Associated Ig-like Receptor-1 (LAIR-1) and produces specialized pro-resolving lipid mediators (SPMs) and promotes M2-like macrophage polarization, which contributes to the resolution of inflammation.

RAGE is an approximately 40 kDa of a pattern recognition receptor belonging to the immunoglobulin superfamily [45]. RAGE is evolutionarily present only in mammals [46], and its deficiency in mice show normal development [47,48]. RAGE is a type I transmembrane protein composed of three extracellular immunoglobulin-like domains (V, C1, and C2), a single transmembrane helix, and a C-terminal short domain, and exists in lipid rafts [45,49] (Figure 2). RAGE has various ligands such as HMGB1, AGEs, S100/calgranulin family, Mac-1, β sheet fibrils, and LPS can bind predominantly with the V domain of RAGE [50,51]. Upon ligand binding, RAGE activates multiple intracellular signaling pathways involving in the small guanine nucleotide triphosphatases (GTPases) ras-related C3 botulinum toxin substrate 1 (Rac1)/cell division control protein 42 (Cdc42), Ras-mediated extracellular signal-regulated kinase 1/2 (ERK1/2), Phosphoinositide 3-kinase (PI3-K)/Akt, stress-activated protein kinase/c-Jun-NH2-terminal kinase (SAPK/JNK), p38 mitogen-activated protein kinase (MAPK), NF-κB and caspases [52]. The RAGE cytoplasmic domain regulates cell signaling and function through binding with adaptor proteins including diaphanous homolog 1 (Diaph1), toll-interleukin 1 receptor domain containing adaptor protein (TIRAP), and myeloid differentiation primary response 88 (MyD88) [51,53]. RAGE is a constitutive multimer on the plasma membrane [54], and such multimers comprise at least four RAGE molecules before ligand binding [51,55]. It seems that the multimerization of RAGE could make ligand multimers possible to bind and sustain downstream signal transduction [51,52]. RAGE also can form heterodimers with other membrane proteins and exert various biological functions. It is reported that DNAX-activating protein 10, a transmembrane protein, also bound to RAGE resulted in an enhancement of Akt activation while homomultimeric RAGE led to the activation of caspase-8 [56] (Figure 3).

RAGE itself contains a tolerogenic system to control overreactions. RAGE has three variants: full length, N-terminally truncated, and C-terminally truncated. Endogenously secreted RAGE, a spliced variant of RAGE, is the C-terminally truncated form of RAGE secreted from the cell and has a V-domain essential for binding ligands [57]. Another isoform, soluble RAGE (sRAGE), is cleaved from cell-surface RAGE by matrix metalloproteinases [58]. Secreted RAGE and truncated RAGE serve as a decoy receptor sequestering ligands and inhibiting signal transduction [57,58,59] (Table 1).

In 1995, Hori et al. first reported that HMGB1 could bind to RAGE [60]. Amino acids 150–183 of HMGB1 are responsible for binding to RAGE for invasive migration and growth of tumor cells through the activation of p38 MAPK and Erk1/2 [61,62]. HMGB1 induced phosphorylation of endogenous RAGE cytosolic domain at Ser391 by PKCζ, and seemed to interact with TIRAP and MyD88, then transduce signals to the downstream molecules such as NF-κB [63]. RAGE is also localized in mitochondria of tumor cells [64], and interaction between HMGB1 and RAGE regulates cellular metabolism and promotes tumor growth by enhancing ATP production. The interaction HMGB1 and RAGE also has been reported to trigger neutrophil-mediated injury amplification following necrosis [65].

Therapeutics targeting RAGE has been developed. Blockade of RAGE signaling by antibody or soluble RAGE also has been shown to the efficacy in several disease models including diabetic complications, sepsis, and autoimmunity [66,67,68,69]. Clinical development may have progressed most in small-molecule inhibitors of RAGE. TTP488, which was discovered by the pharmaceutical industry, is an orally active antagonist of RAGE-RAGE ligand interaction [70] and tested in Phase 2 clinical trials to treat Alzheimer’s disease [71]. FPS-ZM1 was found by screening for molecules inhibiting amyloid β binding to the V domain of RAGE [72]. The efficacy of FPS-ZM1 for cerebral hemorrhage, cerebral ischemia, emphysema, cancer, and inflammatory bowel diseases, have been reported [73,74,75,76,77]. Another molecule inhibiting RAGE signaling is a DNA oligonucleotide aptamer (RAGE-aptamer) [78]. RAGE-aptamer has been reported to reverse and prevent the development of diabetic nephropathy in vivo, and suppressed the AGE-induced reactive oxygen species (ROS) generation and inflammatory and fibrotic reactions in vitro [78]. RAGE-aptamer also attenuates melanoma growth and liver metastasis in vivo by reducing tumor-associated angiogenesis and macrophage infiltration by suppressing the AGE-RAGE system [79].

## 4. The HMGB1-RAGE Axis in SLE

RAGE provides a functional platform for crosstalk with other HMGB1 receptors that exist in organelles. The HMGB1-DNA complexes binding with RAGE on the cell surface result in internalizing into the cytosol and interacting with TLR9, which exist in the endosome, augment type 1 IFN production through a mechanism dependent on MyD88 [80]. Type 1 IFN plays an important role in the pathogenesis of SLE. The DNA-containing immune complexes are also a key factor activation of autoreactive B cells and the induction of type I IFN dependent on RAGE engagement [80]. Further, HMGB1-DNA internalization by RAGE also has been reported in inflammatory monocytes exposed to serum from patients with SLE [81]. Thus, HMGB1 and RAGE are involved in autoimmunity by transmitting intracellular signals and acting as a carrier between the extracellular and intracellular compartments.

Lupus nephritis (LN), which is characterized by renal deposition of immune complexes, is a refractory complication of SLE, which causes end-stage renal disease resulting in lower survival rates and quality of life [6]. It has been reported that anti-DNA antibody binding with HMGB1 exhibited a synergistic proinflammatory effect on mesangial cells of LN patients in a RAGE dependent manner [82]. They also found enhanced susceptibility of lupus prone MRL/lpr mice as compared to normal mice derived mesangial cells to anti-DNA antibody and LPS stimulation, in addition to significantly increased expression of TLR4 [82]. Increased HMGB1 expression deteriorated the severity of SLE via enhancing macrophage inflammatory response, and RAGE played a critical role in HMGB1-mediated macrophage inflammatory response [41]. Treatment with sRAGE, the soluble extracellular domains of RAGE, which blocks ligands interaction with RAGE demonstrated significant improvement of nephritis in (NZB/NZW) F1 lupus-prone mice [69]. They have shown that sRAGE interrupted the nuclear translocation of NF-κB in the kidney, resulting in a reduction in the expression of downstream genes of NF-κB in vivo and in vitro. Moreover, plasma sRAGE level in patients with SLE was significantly lower than those in healthy controls and negatively correlated with SLE disease activity, suggesting a rationale for sRAGE as a therapeutic [83,84]. Though blocking RAGE signaling seems a promising approach to treat LN, there is a controversial report. The RAGE knock-out mice with C57BL/6 background lpr mice exacerbated of autoantibody titers and nephritis was observed [85]. RAGE knock-out lupus mice exhibited a delay in apoptosis of CD3 + B220 + CD4 − CD8 − autoreactive T cells, and an increase in these pathogenic T cells was thought to exacerbate the disease [85].

## 5. The HMGB1-RAGE Axis in Autoimmune Vasculitis

HMGB1 plays an important role in the pathogenic mechanism of autoimmune vasculitis. Like lupus, circulating HMGB1 levels have been reported to be increased and closely associated with the disease activity of AAV [86,87]. HMGB1 could prime neutrophils by increasing ANCA antigens translocation to the cell surface. The primed neutrophils could be further induced by ANCA, resulting in the respiratory burst and degranulation in TLR4- and RAGE-dependent manner through the MyD88/NF-κB pathway [88]. Recently, the abnormal regulation of neutrophil extracellular traps (NETs), generated by ANCA-activated neutrophils, have been recognized to contribute to the pathogenesis of AAV [89,90]. NETs can stick to the endothelium and cause tissue damage during inflammation [91]. It is also reported that NETs are associated with thrombosis in AAV patients as histones and DNA within NETs can bind platelets and blood coagulants [90,92]. HMGB1 exerts effects on NETs formation through interaction with TLR2, TLR4, and RAGE, and the process is NADPH oxidase dependent [93]. Another study suggests that HMGB1 from activated platelets commit neutrophils to NET generation through RAGE, resulting in preventing depletion of mitochondrial potential, autophagosome formation, and prolonging neutrophil survival [94]. Thus, the HMGB1-RAGE axis can potentiate ANCA-inducing NETs formation and may be involved in the pathogenesis of AAV.

Peschel and colleagues have revealed the high prevalence of autoantibodies to lysosome-associated membrane protein-2 (LAMP-2) in ANCA-negative pauci-immune focal necrotizing glomerulonephritis [95]. LAMP-2 is a heavily glycosylated membrane protein that traffics from the cell surface to lysosomes, where it is critical for cellular homeostasis and responses to stress by participating in autophagy [96]. LAMP-2 is an endocytic receptor on human monocyte-derived dendritic cells that routes cargo into immunogenic exosomes while reducing surface expression of antigen-derived peptides [97]. A perspective from immune tolerance of the HMGB1-RAGE axis might provide a novel understanding of the pathogenesis of AAV. There is an association of the HMGB1-RAGE axis with autophagy [98,99].

The anti-inflammatory effects of HMGB1 blockades, including anti-HMGB1 monoclonal antibody and glycyrrhizin, have been shown in a mouse model of cutaneous vasculitis [100].

## 6. The HMGB1-RAGE Axis in Ischemic Diseases

The HMGB1-RAGE axis also involves in the pathophysiology of ischemic diseases. Watanabe et al. first described the relationship between HMGB1 and ischemia-reperfusion (I/R) injury [101]. During hepatic I/R injury, HMGB1 increased and translocated from nuclear to the cytoplasm as early as one hour after ischemia [102]. Inhibition of HMGB1 activity with neutralizing antibody decreased liver damage. In contrast, the administration of recombinant HMGB1 worsened I/R injury [102]. Blood HMGB1 level is also elevated in patients with cerebral or myocardial ischemia. Kim et al. reported the relationship between HMGB1 and brain inflammatory injury in the middle cerebral artery occlusion/reperfusion-induced injury model [103]. HMGB1 also promotes angiogenesis and neurovascular remodeling via endothelial progenitor cells by binding to the RAGE [104].

The HMGB1-RAGE axis also could take part in hypoxia-induced organ damages. Hypoxia induces HMGB1 and AGE accumulation which further formed a complex with RAGE and activates several downstream pathways including NF-κB, hypoxia-inducible factor-1 (HIF-1α), ERK1/2, and Akt signaling [105,106]. It has been demonstrated that the RAGE promoter region contains at least one functional HIF-1 binding site, and HIF-1α down-regulation drastically decreased RAGE induction by hypoxia in neurons, suggesting that hypoxic stimulation of RAGE expression could be mediated by Hif1α [107]. The renal hypoxia promotes ROS formation, and it appears that oxidative stress is a central regulator of HMGB1′s translocation, release, and activity in inflammation and cell death [35]. RAGE-dependent vascular perturbation in hypoxia has also been identified, and the vascular dysfunction may amplify hypoxic HMGB1-RAGE mediated organ damages [108]. Thus, this axis may serve as a master regulator of inflammatory stress triggered by hypoxia.

Dobutamine mediates HO-1 induction via Nrf-2 translocation to inhibit the HMGB1 release in rat myocardial I/R injury. These suggest that Nrf-2-HO-1 axis may serve as a regulator for the HMGB1-RAGE axis and provide a potential therapeutic target in ischemic diseases [109,110].

## 7. Tolerogenic Role of the HMGB1-RAGE Axis

Recent studies demonstrate that several molecules neutralize extracellular HMGB1 or convert its pro-inflammatory functions to anti-inflammatory functions. While the HMGB1-RAGE axis drives inflammation, it also could regulate the immune tolerance by inducing anti-inflammatory macrophages. RAGE could exert various functions by interacting with other membrane receptors and the HMGB1-RAGE axis exerts tolerogenic functions in certain conditions. Under particular microenvironments or with proximal receptors, RAGE can induce immune tolerance. G-protein-coupled receptors involved in the cAMP signal pathway and the phosphatidylinositol signal pathway, interact with RAGE. Mainly, formyl-peptide-receptors play an essential role in amyloid β-induced ERK1/2 phosphorylation and changes in cAMP levels in glial cells by interacting with RAGE [111]. Leukotriene B_4_ (LTB_4_) receptor 1 (BLT1) interacts with RAGE and induces proinflammatory cytokines and chemokines in vitro/ex vivo [112]. Both LTB_4_-dependent ERK1/2 phosphorylation in neutrophils and LTB_4_-dependent neutrophil accumulation in a murine peritonitis model were attenuated in RAGE-deficient mice compared with wild-type mice [112] (Figure 3). Moreover, it has been reported the paradoxical role of HMGB1 in the tumor microenvironment [113]. HMGB1 contributes to the protumoral activities of the M2 macrophage phenotype by a RAGE-dependent mechanism [114]. Released HMGB1 due to hypoxia promotes M2-like macrophage accumulation and an IL-10 rich milieu by selectively signaling through RAGE [106]. We have recently demonstrated that complement component C1q can form a multimolecular signaling complex with HMGB1, RAGE, and Leukocyte-Associated Ig-like Receptor-1 (LAIR-1) in lipid rafts, and suppress inflammation by promoting M2-like macrophage polarization [115] (Table 1). We have also shown that HMGB1 promotes leukotriene production, induces IRF5 in a RAGE-dependent manner, while it produces specialized pro-resolving lipid mediators (SPMs), which have a negative effect on leukotriene synthesis and help in the resolution of inflammation [116,117,118] (Figure 3). These observations indicate that RAGE could not only facilitate inflammation but also resolve inflammation. Thus, the immune homeostasis maintaining the role of RAGE in diverse microenvironments should be examined in future studies. Though further studies are required to elucidate the environmental factors that determine the various function of the HMGB1-RAGE axis, C1q-LAIR-1 is one of the most important partners.

The HMGB1-RAGE axis has a role in regulatory T cells (Tregs), which is a subset of CD4+ T cells and dampening T-cell immune responses against self-antigens and maintaining immunological tolerance. Wild et al. have found that Tregs express significantly higher RAGE levels on the cell surface than conventional T cells, and HMGB1 induces Tregs migration and prolonged their survival in a RAGE-dependent manner [119]. Yang et al. reported that CD163 is an anti-inflammatory receptor for HMGB1-haptoglobin complexes [120]. Haptoglobin, the acute phase protein that binds extracellular hemoglobin and targets cellular uptake through CD163, also binds HMGB1. Haptoglobin is an endogenous HMGB1 binding protein that directs HMGB1 to a CD163-dependent receptor pathway that elicits HO-1 and IL-10 in the monocyte-macrophage lineage [120]. Another study has shown that binding of HMGB1 by soluble CD52, a glycophosphatidylinositol-anchored glycoprotein, promotes ligation of soluble CD52 with the sialic acid-binding Ig-like lectin-10 receptor and suppression of T cell function [121] (Table 1).

**Table 1:** Immune tolerance functions of the HMGB1-RAGE axis.

Molecule	Mode of Action	Reference
Soluble RAGE	Decoy receptor for RAGE	[59]
C1q	Induce anti-inflammatory macrophage polarization	[115,118]
Haptoglobin	Bind with CD163, activates heme oxygenase-1 IL-10 productions	[120]
Soluble CD52	Engage with the sialic acid-binding Ig-like lectin-10 receptor and suppress T cell function	[121]

High mobility group box 1, HMGB1; receptor for advanced glycation endproducts, RAGE.

HMGB1 also demonstrates immunosuppressive activities through myeloid-derived suppressor cells (MDSCs) present in patients with solid tumors and contributes to immune suppression [122]. In murine tumor systems, HMGB1 foments the development of MDSC from bone marrow progenitor cells, enhances crosstalk between MDSC and macrophages by increasing MDSC production of IL-10, and reduces the expression of L-selectin on circulating T cells [123].

## 8. Conclusions

HMGB1 is the best well-characterized DAMPs for more than three decades. The unique feature of HMGB1 may operate in the opposite direction as an alarm signal for the environment. Particular receptors, ligands that are evolutionally closed with HMGB1 may maintain homeostasis as well as immune tolerance. For translating to therapeutics targeting the HMGB1-RAGE axis, not only simple inhibition but other strategy focusing on RAGE partners or individual intracellular molecules might be required due to the diversity of this axis.
